# Transferability of Alzheimer Disease Polygenic Risk Score Across Populations and Its Association With Alzheimer Disease-Related Phenotypes

**DOI:** 10.1001/jamanetworkopen.2022.47162

**Published:** 2022-12-15

**Authors:** Sang-Hyuk Jung, Hang-Rai Kim, Min Young Chun, Hyemin Jang, Minyoung Cho, Beomsu Kim, Soyeon Kim, Jee Hyang Jeong, Soo Jin Yoon, Kyung Won Park, Eun-Joo Kim, Bora Yoon, Jae-Won Jang, Yeshin Kim, Jin Yong Hong, Seong Hye Choi, Young Noh, Ko Woon Kim, Si Eun Kim, Jin San Lee, Na-Yeon Jung, Juyoun Lee, Ae Young Lee, Byeong C. Kim, Soo Hyun Cho, Hanna Cho, Jong Hun Kim, Young Hee Jung, Dong Young Lee, Jae-Hong Lee, Eek-Sung Lee, Seung Joo Kim, So Young Moon, Sang Joon Son, Chang Hyung Hong, Jin-Sik Bae, Sunghoon Lee, Duk L. Na, Sang Won Seo, Carlos Cruchaga, Hee Jin Kim, Hong-Hee Won

**Affiliations:** 1 Department of Biostatistics, Epidemiology and Informatics, Perelman School of Medicine, University of Pennsylvania, Philadelphia; 2 Department of Digital Health, Samsung Advanced Institute for Health Sciences & Technology, Sungkyunkwan University, Samsung Medical Center, Seoul, Republic of Korea; 3 Department of Neurology, Dongguk University Ilsan Hospital, Dongguk University College of Medicine, Goyang, Republic of Korea; 4 Department of Neurology, Samsung Medical Center, Sungkyunkwan University School of Medicine, Seoul, Republic of Korea; 5 Alzheimer’s Disease Convergence Research Center, Samsung Medical Center, Seoul, Republic of Korea; 6 Department of Neurology, Ewha Womans University Seoul Hospital, Ewha Womans University School of Medicine, Seoul, Republic of Korea; 7 Department of Neurology, Eulji University Hospital, Eulji University School of Medicine, Daejeon, Republic of Korea; 8 Department of Neurology, Dong-A University College of Medicine, Department of Translational Biomedical Sciences, Graduate School of Dong-A University, Busan, Republic of Korea; 9 Department of Neurology, Pusan National University Hospital, Pusan National University School of Medicine and Medical Research Institute, Busan, Republic of Korea; 10 Department of Neurology, Konyang University College of Medicine, Daejeon, Republic of Korea; 11 Department of Neurology, Kangwon National University Hospital, Kangwon National University College of Medicine, Chuncheon, Republic of Korea; 12 Department of Neurology, Yonsei University Wonju College of Medicine, Wonju, Republic of Korea; 13 Department of Neurology, Inha University School of Medicine, Incheon, Republic of Korea; 14 Department of Neurology, Gachon University College of Medicine, Gil Medical Center, Incheon, Republic of Korea; 15 Department of Neurology, School of Medicine, Jeonbuk National University Hospital, Jeonju, Republic of Korea; 16 Department of Neurology, Inje University College of Medicine, Haeundae Paik Hospital, Busan, Republic of Korea; 17 Department of Neurology, Kyung Hee University College of Medicine, Kyung Hee University Hospital, Seoul, Republic of Korea; 18 Department of Neurology, Pusan National University Yangsan Hospital, Pusan National University School of Medicine and Medical Research Institute, Busan, Republic of Korea; 19 Department of Neurology, Chungnam National University Hospital, Daejeon, Republic of Korea; 20 Departmet of Neurology, Chonnam National University School of Medicine, Gwangju, Republic of Korea; 21 Department of Neurology, Gangnam Severance Hospital, Yonsei University College of Medicine, Seoul, Republic of Korea; 22 Department of Neurology, National Health Insurance Service Ilsan Hospital, Goyang, Republic of Korea; 23 Department of Neurology, Myongji Hospital, Hanyang University, Goyang, Republic of Korea; 24 Department of Psychiatry, Seoul National University Hospital, Seoul, Republic of Korea; 25 Department of Neurology, University of Ulsan College of Medicine, Asan Medical Center, Seoul, Republic of Korea; 26 Department of Neurology, Soonchunhyang University Bucheon Hospital, Bucheon, Republic of Korea; 27 Department of Neurology, Gyeongsang National University School of Medicine and Gyeongsang National University Changwon Hospital, Changwon, Republic of Korea; 28 Department of Neurology, Ajou University School of Medicine, Suwon, Republic of Korea; 29 Department of Psychiatry, Ajou University School of Medicine, Suwon, Republic of Korea; 30 Eone-Diagnomics Genome Center (EDGC), Incheon, Republic of Korea; 31 Department of Health Sciences and Technology, SAIHST, Sungkyunkwan University, Seoul, Republic of Korea; 32 Department of Intelligent Precision Healthcare Convergence, Sungkyunkwan University, Seoul, Republic of Korea; 33 Department of Psychiatry, Washington University School of Medicine, St Louis, Missouri; 34 NeuroGenomics and Informatics Center, Washington University School of Medicine, St Louis, Missouri; 35 The Charles F. and Joanne Knight Alzheimer Disease Research Center, Washington University School of Medicine, St Louis, Missouri; 36 Samsung Genome Institute, Samsung Medical Center, Seoul, Republic of Korea

## Abstract

**Importance:**

Polygenic risk scores (PRSs), which aggregate the genetic effects of single-nucleotide variants identified in genome-wide association studies (GWASs), can help distinguish individuals at a high genetic risk for Alzheimer disease (AD). However, genetic studies have predominantly focused on populations of European ancestry.

**Objective:**

To evaluate the transferability of a PRS for AD in the Korean population using summary statistics from a prior GWAS of European populations.

**Design, Setting, and Participants:**

This cohort study developed a PRS based on the summary statistics of a large-scale GWAS of a European population (the International Genomics of Alzheimer Project; 21 982 AD cases and 41 944 controls). This PRS was tested for an association with AD dementia and its related phenotypes in 1634 Korean individuals, who were recruited from 2013 to 2019. The association of a PRS based on a GWAS of a Japanese population (the National Center for Geriatrics and Gerontology; 3962 AD cases and 4074 controls) and a transancestry meta-analysis of European and Japanese GWASs was also evaluated. Data were analyzed from December 2020 to June 2021.

**Main Outcomes and Measures:**

Risk of AD dementia, amnestic mild cognitive impairment (aMCI), earlier symptom onset, and amyloid β deposition (Aβ).

**Results:**

A total of 1634 Korean patients (969 women [59.3%]), including 716 individuals (43.6%) with AD dementia, 222 (13.6%) with aMCI, and 699 (42.8%) cognitively unimpaired controls, were analyzed in this study. The mean (SD) age of the participants was 71.6 (9.0) years. Higher PRS was associated with a higher risk of AD dementia independent of *APOE* ɛ4 status in the Korean population (OR, 1.95; 95% CI, 1.40-2.72; *P* < .001). Furthermore, PRS was associated with aMCI, earlier symptom onset, and Aβ deposition independent of *APOE* ɛ4 status. The PRS based on a transancestry meta-analysis of data sets comprising 2 distinct ancestries showed a slightly improved accuracy.

**Conclusions and Relevance:**

In this cohort study, a PRS derived from a European GWAS identified individuals at a high risk for AD dementia in the Korean population. These findings emphasize the transancestry transferability and clinical value of PRSs and suggest the importance of enriching diversity in genetic studies of AD.

## Key Points

**Question:**

Is a polygenic risk score (PRS) derived from European ancestry data associated with Alzheimer disease (AD) dementia risk in non-European populations?

**Findings:**

In this cohort study of genome data from European and Asian databases of patients with AD, a PRS derived from a genome-wide study of individuals with European ancestry was associated with high genetic risk for AD dementia in 1634 Korean individuals. Furthermore, the PRS was associated with amnestic mild cognitive impairment, earlier symptom onset of AD dementia, and amyloid β deposition.

**Meaning:**

These findings emphasize the transancestry transferability and clinical value of PRSs and reinforce the need for enriching diversity in genetic studies of AD.

## Teaser

This cohort study evaluates the ability of a polygenic risk score for Alzheimer disease (AD) created from a genome-wide association study of European populations to assess risk of AD dementia and related outcomes among patients in Korea.

## Introduction

Alzheimer disease (AD) is the main cause of dementia, affecting approximately 50 million individuals worldwide, and the number is expected to triple by 2050 owing to population aging.^1^ This is particularly problematic in East Asia, where the population is aging rapidly. It is estimated that nearly a quarter of patients with dementia live in East Asia, and the number is expected to double over the next 20 years.^2^

The pathological process of AD begins long before the onset of clinical dementia. Therefore, identifying individuals at a high risk for developing AD is of utmost importance for potential preventive and therapeutic strategies.^3^ Genetic information can be used to identify individuals at a high risk for AD because the heritability of AD is estimated to be 60% to 80%.^4^ Previous studies have demonstrated that polygenic risk scores (PRSs), which aggregate the genetic effects of single-nucleotide variants (SNVs) identified in genome-wide association studies (GWASs), can help distinguish individuals at a high genetic risk for AD.^5^

However, previous genetic studies were conducted predominantly in populations of European ancestry. Thus, the generalizability of a PRS to non-European populations remains unknown.^6^ A 2019 study examined the risk assessment capability of European ancestry–derived PRSs in samples of non-European ancestry with various phenotypes.^7^ The PRS for AD derived from European populations was also tested in non-Hispanic Black^8,9^ and Caribbean Hispanic individuals.^10^ However, the performance of PRSs for AD in Asian populations has not yet been evaluated.

Our study aimed to evaluate the transferability of a PRS for AD in the Korean population using summary statistics from a prior large-scale GWAS of European populations.^11^ Moreover, we applied our PRS to determine whether it is associated with risk of amnestic mild cognitive impairment (aMCI), earlier symptom onset, or amyloid β (Aβ) deposition. We also evaluated the PRS based on a GWAS of a Japanese population^12^ and a transancestry meta-analysis of European and Japanese GWASs.

## Methods

All participants provided written informed consent in the primary Korean data set, and the study was approved by the institutional review board of each center. This study followed the reporting requirements of the Strengthening the Reporting of Observational Studies in Epidemiology (STROBE) Statement.

### Data Set 1

**Figure 1.**
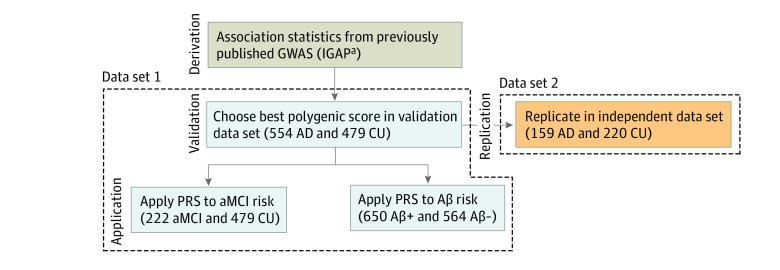
Study Data Sets and Analysis Steps Aβ indicates amyloid β; AD, Alzheimer disease; aMCI, amnestic mild cognitive impairment; CU, cognitively unimpaired; GWAS, genome-wide association study; IGAP, International Genomics of Alzheimer Project. ^a^Summary statistics were obtained from the European GWAS (IGAP).

A total of 1255 participants of Korean ancestry were recruited from 14 referral hospitals in the Republic of Korea from January 2013 to July 2019 (Figure 1). Among them, 954 participants were recruited from the Samsung Medical Center, 202 from a multicenter study of the Korean Brain Aging Study for Early Diagnosis and Prediction of AD,^13^ and 99 from a multicenter clinical research platform study based on the dementia cohort. We included participants who were diagnosed with AD dementia or aMCI or those who were cognitively unimpaired (CU) based on detailed neuropsychological test results.^14,15,16^ We used the participants’ diagnoses at the latest assessment point. AD dementia was defined in accordance with the core clinical criteria for probable AD dementia according to the National Institute on Aging-Alzheimer Association.^15^ aMCI was defined in accordance with the following criteria, modified from Peterson’s criteria^17^: (1) normal activities of daily living performance, (2) objective memory impairment on a verbal or visual memory test below the 16th percentile of age- and education-matched norms, and (3) no dementia.

Participants were excluded when they had (1) a causative genetic mutation for AD in known genes, such as *PSEN1*, *PSEN2*, or *APP*; (2) structural abnormalities detected on brain magnetic resonance imaging, such as severe cerebral ischemia, territorial infarction, or brain tumors; or (3) other medical or psychiatric diseases that may cause cognitive impairment.

### Data Set 2

For the replication data set, 379 participants of Korean ancestry were recruited from 20 referral hospitals in the Republic of Korea. Of these, 125 participants were from the biobank of the Chronic Cerebrovascular Disease Consortium, recruited from 2016 to 2018. This was part of the ongoing BICWALZS study (Biobank Innovation for Chronic Cerebrovascular Disease With Alzheimer’s Disease Study) and data from the Center for Convergence Research of Neurological Disorders.^18^ The remaining 254 participants were recruited from the PREMIER (Precision Medicine Platform for Mild Cognitive Impairment Based on Multi-omics, Imaging and Evidence-based Research & Business Development) study. We included participants who were diagnosed with AD dementia or CU according to the same criteria in data set 1.

### Genotyping and Imputation

DNA samples were genotyped using the Asian screening array (ASA) chip (Illumina). A subset of 125 samples was genotyped using a customized Korea Biobank array (KBA) chip (Affymetrix).^19^ Quality control for SNV data was conducted using the PLINK software and imputation was conducted using the Minimac4 software at the University of Michigan Imputation Server (eMethods in the Supplement).

### Amyloid Positron Emission Tomography (PET)

A subset of 1214 participants in data set 1 underwent either 18F-florbetaben or 18F-flutemetamol PET (eMethods in the Supplement).^20^ Aβ positivity was determined by visual assessments.

### GWAS Summary Statistics

To investigate the transferability of the PRS in the Korean population, we utilized the summary statistics generated from the European International Genomics of Alzheimer Project (IGAP) GWAS (11 480 632 SNVs from 21 982 AD cases and 41 944 controls)^11^ and East Asian–based National Center for Geriatrics and Gerontology (NCGG) Japanese GWAS (4 852 957 SNVs from 3962 AD cases and 4074 controls).^12^ Furthermore, we derived the PRS using transancestry meta-GWAS results (12 519 321 SNVs) obtained from an inverse variance-weighted fixed-effects meta-analysis of the European and Japanese GWAS results using METAL.^21^

### PRS Generation

Based on previous study data,^5,22,23^ we excluded 3877 SNVs surrounding *APOE* (chromosome 19, 44 400 to 46 500 kb, GRCh37/hg19) to derive the PRS independent of the *APOE* region (eMethods in the Supplement). PRSice-2 software version 2.3.3 (GNU General Public License) was used to generate the PRS for AD dementia using prior GWAS summary statistics (European GWAS, Japanese GWAS, or meta-analysis).

### Validation and Replication of the PRS for AD

After calculating each participant’s PRS, we performed a logistic regression analysis to determine whether the PRS derived from the summary statistics for the AD risk based on European populations was associated with AD dementia diagnosis in the data set 1 and 2 after adjusting for age, sex, education year, *APOE* ɛ4 carrier status, and the first 4 principal components of genetic ancestry. To verify whether the association of the PRS with AD dementia diagnosis varied by the *APOE* ɛ4 carrier status, we performed the same analysis after stratifying the participants into *APOE* ɛ4 carriers and noncarriers. In addition, we developed the PRS based on previous Japanese GWAS and transancestry meta-GWAS results and evaluated the association of PRS with AD.

### Application of the PRS in Various Phenotypes

A multivariable logistic regression analysis was conducted for the participants with aMCI to evaluate whether the PRS is associated with aMCI independent of age, sex, education year, *APOE* ɛ4 carrier status, and the first 4 principal components of genetic ancestry.

We stratified the participants based on quartiles of the PRS and evaluated whether the PRS can be used for risk stratification in addition to *APOE* ɛ4 genotyping. We also evaluated whether the participants with a high PRS showed earlier development of AD than did those with a low PRS. We performed a Cox regression analysis with age at AD onset and age at the last clinical visit as time variables and the diagnosis of AD as a status variable.

Furthermore, using a subset of 1214 participants who also underwent Aβ PET, we also performed a logistic regression analysis to evaluate whether the PRS is associated with Aβ positivity. We adjusted for the effect of age at which Aβ PET was performed, sex, education year, and *APOE* ɛ4 carrier status.

### Statistical Analysis

For demographic and clinical characteristics, categorical and continuous variables were presented as totals and mean averages, respectively. The χ^2^ test was used for categorical variables and analysis of variance for continuous variables. Cochran-Armitage tests were used to determine *P* values for trend. We reported 2-tailed *P* values and defined *P* < .05 as statistically significant. All statistical analyses and result visualization were performed using PLINK version 1.90,^24^ R version 3.6.1 (R Project for Statistical Computing), and MATLAB.

## Results

### Participants

Data set 1 included a total of 1255 participants with a mean (SD) age of 72.2 (8.9) years (739 women [58.9%]) (Table 1). Data set 2 included 379 participants with a mean (SD) age of 69.8 (9.3) years (230 women [60.7%]). In the principal component analysis (PCA) with data from the 1000 Genomes Project, there was an ethnic overlap of our data set with those of other East Asian populations. In the East Asian population, mean (5-SD) of PC_1_ and PC_2_ were 0.152 (0.001) and 0.034 (0.005) respectively. In our study cohorts, mean (5-SD) of PC_1_ and PC_2_ were 0.151 (0.001) and 0.032 (0.005), respectively. However, there was no stratification by genotyping arrays (ASA and KBA) (eFigure 1 in the Supplement). In addition, the PRS distributions among the study participants were not significantly different according to the genotyping arrays. In patients with AD dementia, mean (standard error [SE]) of PRS was 0.269 (0.016) and 0.285 (0.051) for those using ASA and KBA chips respectively (*P* = .33). In patient who were CU, mean (SE) of PRS was 0.158 (0.016) and 0.212 (0.053) for those using ASA and KBA chips respectively (*P* = .77) (eFigure 2 in the Supplement).

**Table 1.** Demographic and Clinical Characteristics of the Study Data Sets

Characteristics	Patients, No. (%)				
	Data set 1 (n = 1255)			Data set 2 (n = 379)	
	CU (n = 479)	AD dementia (n = 554)	aMCI (n = 222)	CU (n = 220)	AD dementia (n = 159)
Age, mean (SD), y	70.7 (7.6)	73.1 (10.0)	73.0 (8.2)	67.8 (9.2)	72.6 (8.6)
Sex					
Women	282 (58.9)	348 (62.8)	109 (49.1)	139 (63.2)	91 (57.2)
Men	197 (41.1)	206 (37.2)	113 (50.9)	81 (26.8)	68 (42.8)
Education, mean (SD), y	11.2 (4.9)	10.4 (5.0)	11.9 (4.7)	11.3 (4.6)	9.7 (5.3)
*APOE* ε4 carrier	118 (24.6)	314 (56.7)	79 (35.6)	55 (25.0)	74 (46.5)

Abbreviations: AD, Alzheimer disease; aMCI, amnestic mild cognitive impairment; CU, cognitively unimpaired.

### Optimal PRS Generation for the Korean Population

**Figure 2.**
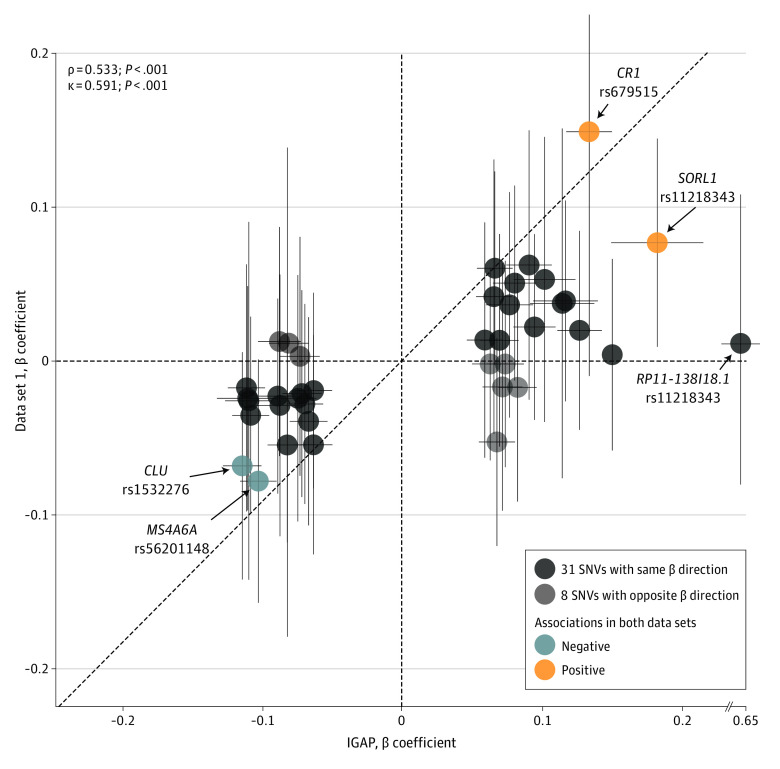
Scatter Plot of β Coefficients of 39 SNVs GWAS indicates genome-wide association study; IGAP, International Genomics of Alzheimer Project; SNV, single-nucleotide variant. Correlations were determined using Spearman correlation (ρ) and Cohen κ coefficient tests between the European GWAS (IGAP) and the data set 1. Detailed information of SNVs shown in eTable 1 in the Supplement.

To determine the best parameters (*P* value threshold cut-off and linkage disequilibrium [LD]-based clumping value) for PRS calculation, we used PRSice-2 using the European GWAS (IGAP) summary statistics. Among various thresholds, we observed the highest Nagelkerke *R*^2^ value (0.020) when the *P* and LD values were 4.15 × 10^−6^ and 0.1, respectively (eFigure 3A in the Supplement). From these thresholds, 39 SNVs were selected, and their β coefficients were used to create the PRS (eTable 1 in the Supplement). We observed a significant correlation between the β coefficients of the 39 SNVs calculated from the European GWAS (IGAP) and those from data set 1 (Spearman correlation = 0.533; *P* < .001) (Figure 2).

### Association of the PRS With AD Dementia, aMCI, and Aβ Deposition

A higher PRS was associated with an increased risk of AD dementia after adjusting for the effect of age, sex, education, and *APOE* ɛ4 status (odds ratio [OR], 1.95; 95% CI, 1.40-2.72; *P* < .001) (Table 2). Furthermore, PRS was also associated with the AD dementia risk in both *APOE* ɛ4 carriers (OR, 2.73; 95% CI, 1.53-4.97; *P* = .001) and noncarriers (OR, 1.70; 95% CI, 1.14-2.59; *P* = .01). These results were replicated in data set 2 (eg, AD dementia diagnosis: OR, 1.85; 95% CI, 1.05-3.32) (Table 2). Similarly, we observed that a higher PRS was significantly associated with an increased risk of aMCI (OR, 1.74; 95% CI, 1.16-2.64; *P* = .008) and Aβ deposition in the brain (OR, 1.81; 95% CI, 1.32-2.48; *P* < .001).

**Table 2.** Association of the PRS With AD Dementia, aMCI, and Aβ Deposition

Measure	OR (95% CI)	*P* value
AD dementia diagnosis^a^		
Data set 1	1.95 (1.40-2.72)	<.001
Data set 2	1.85 (1.05-3.32)	.04
aMCI diagnosis^b^	1.74 (1.16-2.64)	.008
Aβ PET deposition^c^	1.81 (1.32-2.48)	<.001

Abbreviations: Aβ, amyloid β; AD, Alzheimer disease; aMCI, amnestic mild cognitive impairment; CU, cognitively unimpaired; OR, odds ratio; PC, principal component; PET, positron emission tomography; PRS, polygenic risk score.

^a^ Diagnosis (CU = 0, AD dementia = 1) = sex + age + education year + PC_1-4_ + *APOE* ε4 carrier (0 or 1) + PRS.

^b^ Diagnosis (CU = 0, aMCI = 1) = sex + age + education year + PC_1-4_ + *APOE* ε4 carrier (0 or 1) + PRS.

^c^ Aβ deposition (negative = 0, positive = 1) = sex + age + education year + PC_1-4_ + *APOE* ε4 carrier (0 or 1) + PRS.

### Utility of the PRS in Risk Stratification of AD Dementia–Related Outcomes

**Figure 3.**
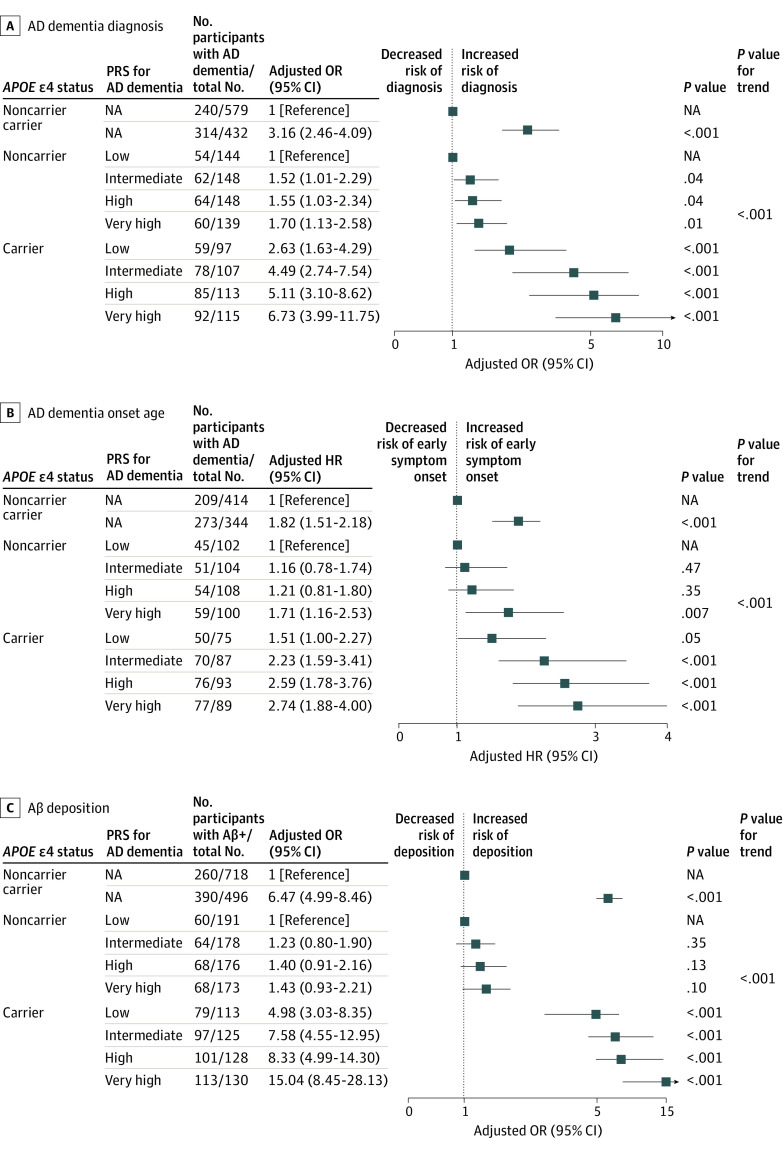
Forest Plots of AD Dementia–Related Outcomes According to the PRS Group and *APOE* ε4 Status Aβ indicates amyloid β; AD, Alzheimer disease; HR, hazard ratio; OR, odds ratio; PRS, polygenic risk score. Adjustments made for the effect of sex, age, education year, and the first 4 principal components from genotyping data. *P* values for trend were determined using Cochran-Armitage tests.

To evaluate the AD dementia risk using the PRS, we stratified the participants according to PRS quartiles. There were significant differences among the PRS risk groups in amyloid positivity, diagnosis, and age at symptom onset. Particularly, the mean age at symptom onset was approximately 3.7 years younger in the very high PRS group than in the low PRS group (mean [SD] of age at symptom onset: low PRS group, 69.0 [9.9] vs very high PRS group, 65.3 [9.7]) (eTable 2 in the Supplement). When we combined PRS and *APOE* ε4 status, we observed a stepwise increase in the risk of AD dementia, earlier age at symptom onset, and Aβ deposition according to the PRS quartile in both *APOE* ε4 carriers and noncarriers (Figure 3). Notably, compared with *APOE* ε4 noncarriers in the low PRS group, the *APOE* ε4 carriers in the very high PRS group showed a 6.73-fold (95% CI, 3.99-11.75), 2.74-fold (95% CI, 1.88-4.00), and 15.04-fold (95% CI, 8.45-28.13) higher risk for AD dementia, earlier age at symptom onset, and Aβ deposition, respectively.

### Transferability of the PRS Based on the Japanese GWAS and Meta-analysis GWAS Data

We further evaluated the PRS derived from the Japanese GWAS (3962 AD cases and 4074 controls). Across the various thresholds (*P* and LD values), the highest Nagelkerke *R*^2^ value for PRS was 0.006 (*P* = .03) when the *P* and LD values were 5.00 × 10^−8^ and 0.1, respectively, which was smaller than PRS based on the European GWAS (Nagelkerke *R*^2^ = 0.020; *P* < .001). Next, we performed transancestry meta-GWAS from European and Japanese GWAS (eFigure 4 in the Supplement). When we developed PRS from the transancestry meta-GWAS, the transancestry PRS achieved the highest performance (Nagelkerke *R*^2^ = 0.023; *P* < .001) among other PRSs (eFigure 3, eTables 3 and 5 in the Supplement). In contrast to European population–based and transancestry PRS, Japanese population–based PRS showed the highest performance when using a single SNV (eTable 4 in the Supplement) and its estimation of AD dementia risk was not replicated in the data set 2 (eTable 5 in the Supplement).

## Discussion

In this study, we demonstrated that a PRS derived from a prior GWAS of European ancestry was associated with AD dementia risk independent of *APOE* ɛ4 status in the Korean population. Furthermore, the PRS was associated with aMCI, earlier symptom onset of AD dementia, and Aβ deposition independent of *APOE* ɛ4 status.

Our results support the potential utility of a prior large-scale GWAS of one population in developing a PRS in another population. Consistent with our findings, previous studies have shown that a PRS for AD derived from European GWASs accurately estimated the dementia risk among non-Hispanic Black^8,9^ and Caribbean Hispanic individuals.^10^ In our multivariable logistic model, PRS was associated with the AD dementia risk independent of the *APOE* ɛ4 carrier status, which was replicated in the independent data set. We observed a stepwise increase in the risk of AD dementia with increased PRS quantiles (Figure 3). In our Korean population data set, the *APOE* ɛ4 status showed the highest effect size among the factors, including the PRS, confirming that the *APOE* ɛ4 status is an important risk factor for AD dementia across various ancestries.^25^ However, risk stratification based on the *APOE* ɛ4 status alone might be insufficient because individuals are classified into only 3 genotypes (*APOE* ɛ4 noncarrier, heterozygous carrier, and homozygous carrier), and this status does not provide sufficient explanation of the phenotypic variance of AD. In this regard, aside from the *APOE* genotype, PRS may further explain the phenotypic variance and represent the polygenicity of AD dementia. Notably, compared with *APOE* ε4 noncarriers in the low PRS group, the *APOE* ε4 carriers in the very high PRS group showed a 6.73-fold (95% CI, 3.99-11.75) higher risk for AD dementia. Therefore, PRS is expected to be a useful risk tool for assessing risk in addition to the *APOE* ɛ4 status in precision medicine.

We demonstrated that higher PRS was associated with increased risk of aMCI. This is consistent with previous findings that PRS for AD was associated with MCI.^26,27^ In addition, we observed that patients with higher PRS were more likely to develop AD dementia symptoms at a younger age. The mean age at symptom onset was approximately 3.7 years younger in the very high PRS group compared with the low PRS group. It is well known that *APOE* ɛ4 is associated with earlier symptom onset of AD dementia. Our results showed that PRS further indicates an acceleration in the age at symptom onset beyond the effect of *APOE* ɛ4. A previous study also showed that PRS derived from 23 genetic variants was associated with the age at symptom onset of AD dementia.^28^

Furthermore, we found a significant association between PRS and Aβ positivity independent of the *APOE* ɛ4 carrier status (Figure 3). When we stratified participants according to *APOE* genotype and PRS, we found that compared with *APOE* ε4 noncarriers in the low PRS group, the *APOE* ε4 carriers in the very high PRS group showed a 15.04-fold (95% CI, 8.45-28.13) higher risk for Aβ deposition (Figure 3). This is in line with previous findings,^22,29,30,31,32,33,34^ which showed that the PRS was associated with AD pathologies (Aβ deposition, τ, and neurodegeneration). Identifying patients with Aβ deposition is crucial in predicting prognosis and selecting patients for clinical trials of anti-Aβ therapy.^35^ Currently available diagnostic tools for measuring Aβ deposition are either invasive (cerebrospinal fluid examination) or expensive (PET).^36^ Our findings highlight that the genetic data (PRS and *APOE* ɛ4 status) obtained from less invasive methods (blood or saliva specimen evaluation) can be used to prescreen individuals for Aβ positivity. These findings indicate the potential use of the PRS to promote early intervention by early identification of individuals at an increased risk for AD dementia.

The performance of the PRS was low when it was developed based on a prior GWAS of the Japanese population (eFigure 3 in the Supplement), despite the closer genetic relatedness of Korean individuals with Japanese populations than with European populations, as shown in the 1000 Genomes Project data set (eFigure 1 in the Supplement). We speculated that this low performance could be attributed to the difference in the sample size of the GWAS (8036 Japanese patients vs 63 926 European patients). The GWAS with a larger sample size identified more SNVs, estimated more accurate β coefficients of each SNV, and further improved the performance of PRS compared with its counterpart. When we used the data from the transancestry meta-analysis of European and Japanese GWAS, the transancestry PRS achieved the highest Nagelkerke *R*^2^ value for AD dementia in the Korean population, indicating the importance of ancestral background as well. As an additional point of view, several studies have shown that different LD patterns could affect the transferability when a risk is assessed by tagged SNVs from different ancestral backgrounds.^37,38,39,40^ Thus, the sample size and ancestral background of prior GWASs are both important factors in developing PRS.

### Limitations

This study has several limitations. First, the sample size of the Korean population was relatively small compared with that of the European population, which limited the statistical power to compare the effects of each variant between populations. Although this study was performed in thoroughly phenotyped subjects using clinical and neuroimaging data, our findings should be replicated in larger independent data sets. Second, the findings of this study were limited to the Korean population. Subsequent studies including other East Asian populations, such as Chinese or Japanese populations, may further strengthen the evidence for transancestry transferability of PRS in the East Asian population.

## Conclusions

This cohort study found that a PRS derived from a European GWAS was associated with AD dementia independent of *APOE* ɛ4 status in the Korean population. Furthermore, it was associated with aMCI, earlier symptom onset of AD dementia, and Aβ deposition independent of *APOE* ɛ4 status. Our findings emphasize the ancestral transferability and clinical value of the PRS and further emphasizes the need for enriching diversity in genetic studies of AD.

## Notes

Supplement.
eMethods.
**eTable 1.** Thirty-nine SNVs Used in the Best-fit PRS **eTable 2.** Demographics of the Participants According to the PRS Quantiles **eTable 3.** Characteristics of SNVs Selected for Transancestry PRS **eTable 4.** Characteristics of SNVs Selected for Japanese-based PRS **eTable 5.** Predictive Accuracy of PRSs Derived From European, Japanese, and Transancestry Meta-GWAS **eFigure 1.** Principal Component Analysis Comparison of the Korean Study Cohorts and the 1000 Genomes Project Populations **eFigure 2.** PRS Distributions Among the Study Participants According to the Genotyping Arrays **eFigure 3.** Distribution Plots for Nagelkerke’s *R*^2^ Values of Each Population-based PRS Across the SNV Selection Thresholds **eFigure 4.** Quantile-quantile Plot and Miami Plot for the Transancestry Meta-GWAS
eReferences.
