# Supplementary material for: Transferability of Alzheimer Disease Polygenic Risk Score Across Populations and Its Association With Alzheimer Disease-Related Phenotypes
Source: JAMA Netw Open. 2022 Dec 15;5(12):e2247162. doi: 10.1001/jamanetworkopen.2022.47162 (PMC9856322; doi:10.1001/jamanetworkopen.2022.47162)
Supplement: Supplement. — eMethods. eTable 1. Thirty-nine SNVs Used in the Best-fit PRS eTable 2. Demographics of the Participants According to the PRS Quantiles eTable 3. Characteristics of SNVs Selected for Transancestry PRS eTable 4. Characteristics of SNVs Selected for Japanese-based PRS eTable 5. Predictive Accuracy of PRSs Derived From European, Japanese, and Transancestry Meta-GWAS eFigure 1. Principal Component Analysis Comparison of the Korean Study Cohorts and the 1000 Genomes Project Populations eFigure 2. PRS Distributions Among the Study Participants According to the Genotyping Arrays eFigure 3. Distribution Plots for Nagelkerke’s R2 Values of Each Population-based PRS Across the SNV Selection Thresholds eFigure 4. Quantile-quantile Plot and Miami Plot for the Transancestry Meta-GWAS eReferences. [file jamanetwopen-e2247162-s001.pdf]

Supplemental Online Content

Jung S-H, Kim H-R, Chun MY, et al. Transferability of Alzheimer disease polygenic risk score across populations and its association with Alzheimer disease-related phenotypes. *JAMA Netw Open.* 2022;5(12):e2247162. doi:10.1001/jamanetworkopen.2022.47162

eMethods.

eTable 1. Thirty-nine SNVs Used in the Best-fit PRS

eTable 2. Demographics of the Participants According to the PRS Quantiles

eTable 3. Characteristics of SNVs Selected for Transancestry PRS

eTable 4. Characteristics of SNVs Selected for Japanese-based PRS

eTable 5. Predictive Accuracy of PRSs Derived From European, Japanese, and Transancestry Meta-GWAS

eFigure 1. Principal Component Analysis Comparison of the Korean Study Cohorts and the 1000 Genomes Project Populations

eFigure 2. PRS Distributions Among the Study Participants According to the Genotyping Arrays

eFigure 3. Distribution Plots for Nagelkerke's R^2 Values of Each Population-based PRS Across the SNP Selection Thresholds

eFigure 4. Quantile-quantile Plot and Miami Plot for the Transancestry Meta-GWAS

eReferences.

This supplemental material has been provided by the authors to give readers additional information about their work.

© 2022 Jung S-H et al. *JAMA Network Open.*

eMethods.

eMethod 1. Genotyping, imputation, and quality control criteria.

Quality control (QC) was performed for both types of single-nucleotide polymorphism (SNP) data. SNVs were removed using the following criteria: (i) call rate of <98%, (ii) minor allele frequency (MAF) of <1%, or (iii) genotype frequencies significantly deviating from the Hardy–Weinberg equilibrium with a P value of $<10^{-6}$. After QC, the genotype data were imputed to estimate genotypes for variants that were not directly genotyped and to combine datasets of different genotyping arrays (ASA chip and KBA chip). Imputation was conducted using the Minimac4 software with all available reference haplotypes from the Haplotype Reference Consortium (HRC-r1.1 2016) at the University of Michigan Imputation Server. Consequently, we performed post-imputation QC with (i) an MAF of <1% or (ii) a low imputation quality ($R^2<0.8$ for imputed SNVs). To verify the appropriate combination of the two genotype datasets, we performed principal component analysis (PCA) using EIGENSTRAT [1]. We also conducted PCA for 1000 Genomes Project samples, and projected the two genotyped datasets to the PCA plot to confirm the ancestral distinction.

Based on the genotype data, participants were excluded in accordance with the following criteria: (i) call rate of <95%, (ii) sex mismatch, (iii) heterozygosity excess (±5 standard deviations from the mean), or (iv) one of the related pairs of individuals with second-degree or closer relationships estimated using the KING software [2].

© 2022 Jung S-H et al. *JAMA Network Open.*

eMethod 2. Amyloid positron emission tomography acquisition.

Aβ PET images were obtained using a Discovery STE PET/computed tomography scanner (GE Medical Systems, Milwaukee, WI, USA). PET images were acquired for 20 min, starting at 90 min after intravenous injection of either 18F-florbetaben or 18F-flutemetamol. Aβ positivity or negativity was determined by well-trained nuclear physicians using visual assessments for florbetaben or flutemetamol PET [3, 4]. Positivity for tracer uptake was assessed in four cortical regions (lateral temporal, frontal, parietal, and posterior cingulate cortices) for florbetaben PET and five regions (lateral temporal, frontal, parietal, posterior cingulate cortices, and striatum) for flutemetamol PET. Amyloid PET positivity was defined as having at least one cortical region with evidence of a positive uptake.

© 2022 Jung S-H et al. *JAMA Network Open.*

eMethod 3. Polygenic risk score generation.

To generate the best polygenic risk score (PRS) model *P* values and effect sizes from the summary statistics were used in the dataset 1 (Korean population of 554 Alzheimer's disease dementia (ADD) cases and 479 cognitively unimpaired (CU) controls). Briefly, the PRS for each participant was generated from the sum of the effect sizes from all associated alleles included in the best model. To derive the best model, we tested the inclusion of SNVs from a range of *P* value thresholds (5×10^{-8}–1.0) from the genome-wide association study conducted by Kunkle et al [5]. Further, we tested a range of linkage disequilibrium-based clumping r2 (0.1–0.9) within 1,000 kb to examine which thresholds resulted in the largest Nagelkerke's R^2 value, calculated from the logistic regression. Subsequently, we used the same SNVs and weights to replicate the association of the PRS in a dataset 2 of 379 samples (159 ADD cases and 220 CU controls) and in the dataset 1 of 222 patients with amnestic mild cognitive impairment. The overview of the study is shown in Fig. 1.

© 2022 Jung S-H et al. *JAMA Network Open.*

eTable 1. Thirty-nine SNVs used in the best-fit PRS.

CHR	SNP	Nearest gene	EA	European[1]	East Asian[1]	European GWAS (IGAP) [5]		Korean (our dataset 1)	
				EAF	EAF	Beta[2]	SE[2]	Beta[3]	SE[3]
10	rs12358692	*RP11-138I18.1*	T	0.6572	0.3264	0.6429	0.0154	0.0133	0.0828
11	rs11218343	*SORL1*	T	0.9611	0.7023	0.2053	0.0369	0.0936	0.0826
2	rs6733839	*BIN1*	T	0.3921	0.4363	0.1693	0.0154	0.0048	0.0759
1	rs679515	*CR1*	T	0.1988	0.0284	0.1508	0.0183	0.1816	0.2186
1	rs1752684	*CR1*	A	0.2037	0.3526	0.1432	0.0178	0.0241	0.0789
20	rs6014724	*CASS4*	A	0.9095	0.6311	0.1319	0.0259	0.0474	0.0796
3	rs6805148	*CLEC3B*	A	0.9109	0.9188	0.1293	0.0257	0.0455	0.1387
8	rs1532276	*CLU*	T	0.3919	0.2031	-0.1266	0.0154	-0.0834	0.0901
11	rs1582763	*MS4A6A*	A	0.3593	0.1330	-0.1232	0.0149	-0.0215	0.0980
14	rs17125924	*FERMT2*	A	0.0954	0.2262	0.1222	0.0246	0.0298	0.0889
2	rs35832505	*BIN1*	T	0.1662	0.0562	0.1213	0.019	0.0318	0.1419
11	rs3851179	*PICALM*	T	0.3542	0.4103	-0.1198	0.0148	-0.0433	0.0783
2	rs35695568	*RPL21P32*	T	0.0963	0.1247	0.1152	0.0247	0.0645	0.1130
11	rs56201148	*MS4A6A*	T	0.3823	0.2189	-0.1137	0.0146	-0.0955	0.0965
19	rs12151021	*ABCA7*	A	0.6795	0.5081	-0.1071	0.0169	-0.0267	0.0737
7	rs11767557	*EPHA1-AS1*	T	0.7989	0.1401	0.1028	0.0182	0.0759	0.1068
11	rs67472071	*SPI1*	A	0.3396	0.3026	-0.0981	0.0152	-0.0281	0.0773
19	rs3795065	*ABCA7*	T	0.3498	0.2548	0.0968	0.0171	-0.0152	0.0908
16	rs3752786	*MTSS2*	A	0.2213	0.1324	0.0964	0.0209	0.0354	0.1038
8	rs73223431	*PTK2B*	T	0.3352	0.2991	0.0936	0.0153	-0.0211	0.0906
14	rs11623019	*SLC24A4*	T	0.8066	0.5553	-0.0913	0.0174	-0.0615	0.0773
14	rs12590654	*SLC24A4*	A	0.3429	0.4640	-0.0906	0.0157	-0.0668	0.0772
11	rs11039165	*MADD*	A	0.2668	0.0244	0.0894	0.0158	-0.0139	0.2328
16	rs28482811	*GPRC5B*	T	0.8400	0.7329	0.0872	0.0189	0.0443	0.0894
6	rs3135348	*BTNL2*	A	0.4198	0.3705	0.0837	0.0150	-0.0026	0.0818
6	rs9381563	*AL355353.1*	T	0.3550	0.1979	0.0821	0.0148	0.0298	0.0977
6	rs9268112	*TSBP1-AS1*	A	0.3256	0.2629	0.0815	0.0159	-0.0208	0.0981
19	rs3865444	*SIGLEC22P*	A	0.3212	0.1733	-0.0804	0.0158	0.0035	0.0947

© 2022 Jung S-H et al. *JAMA Network Open.*

11	rs11230227	*MS4A4E*	A	0.3804	0.2584	0.0792	0.0153	0.0162	0.0843
6	rs9271375	*HLA-DRB1*	A	0.4850	0.6226	-0.0789	0.0157	-0.0261	0.0820
11	rs2293579	*PSMC3*	A	0.3868	0.3743	0.0771	0.0145	-0.0644	0.0825
8	rs7831810	*GULOP*	A	0.5860	0.4013	-0.0765	0.0146	-0.0343	0.0793
5	rs11168036	*PFDN1*	T	0.4918	0.4339	0.0754	0.0143	0.0734	0.0768
11	rs598561	*SLC25A1P1*	A	0.4873	0.0963	0.0747	0.0143	0.0508	0.1088
21	rs3017432	*ADAMTS1*	T	0.6033	0.2664	0.0735	0.0151	0.0481	0.0825
17	rs2526378	*BZRAP1*	A	0.5449	0.4696	-0.0717	0.0145	0.0026	0.0764
19	rs8111708	*ELL*	A	0.3562	0.2523	0.0696	0.0151	0.0667	0.0869
7	rs7805776	*EPHA1-AS1*	A	0.5248	0.3732	-0.0695	0.0148	-0.0237	0.0776
6	rs12197146	*CD2AP*	T	0.5127	0.8269	0.0674	0.0144	0.0164	0.0933

[1]EAF for non-Finnish EUR and EAS samples from the Genome Aggregation Database (gnomAD version 2.1.1, https://gnomad.broadinstitute.org)

Statistical values were obtained from [2]a previous study [5] and [3]our dataset 1.

Abbreviations: IGAP, International Genomics of Alzheimer's Project; CHR, chromosome; SNP, single-nucleotide polymorphism; SE, standard error; EA, effective allele; EAF, effective allele frequency; PRS, polygenic risk score

© 2022 Jung S-H et al. *JAMA Network Open.*

eTable 2. Demographics of the participants according to the PRS quantiles.

	Low PRS group (n=314)	Intermediate PRS group (n=314)	High PRS group (n=314)	Very high PRS group (n=313)	*P*
**Age, mean (SD), y**	72.6 (8.9)	72.6 (8.8)	71.9 (9.1)	71.7 (8.7)	.15
**Education, mean (SD), y**	11.2 (4.9)	10.9 (5.0)	10.7 (5.1)	11.1 (4.9)	.76
**Sex**					.22
**Female, No. (%)**	172 (54.8)	182 (58.0)	197 (62.7)	188 (60.1)	
**Male, No. (%)**	142 (45.2)	132 (42.0)	117 (37.3)	125 (39.9)	
***APOE* ε4 carrier, No. (%)**	117 (37.3)	129 (41.1)	132 (42.0)	133 (42.5)	.53
**Amyloid positivity, No. (%)**	139/304 (45.7)	161/303 (53.1)	169/304 (55.6)	181/303 (59.7)	.005
**Age at ADD symptom onset, mean (SD), y**	69.0 (9.9)	68.1 (9.8)	66.5 (10.4)	65.3 (9.7)	.01
**Diagnosis, No. (%)**					.005
**CU, No. (%)**	150 (47.8)	115 (36.6)	112 (35.7)	102 (32.6)	
**aMCI, No. (%)**	51 (16.2)	59 (18.8)	53 (16.9)	59 (18.8)	
**ADD, No. (%)**	113 (36.0)	140 (44.6)	149 (47.5)	152 (48.6)	

P values were obtained using the chi-square test for categorical variables and analysis of variance for continuous variables.

Abbreviations: CU, cognitively unimpaired; aMCI, amnestic mild cognitive impairment; ADD, Alzheimer's disease dementia; PRS, polygenic risk score; SD, standard deviation.

© 2022 Jung S-H et al. *JAMA Network Open.*

eTable 3. Characteristics of SNVs selected for trans-ancestry PRS.

CHR	SNP	Nearest gene	EA	**Trans-ancestry meta-GWAS**		**European GWAS (IGAP) [5]**			**Japanese GWAS (NCGG) [6]**			**Korean (our dataset 1)**	
				Beta[1]	SE[1]	Beta[2]	SE[2]	[4]Proportion of SNP's weight	Beta[2]	SE[2]	[4]Proportion of SNP's weight	Beta[3]	SE[3]
16	rs56983910	*UNGP1*	T	-0.3818	0.0915	-0.3818	0.0889	100.00%	-	-	-	-0.1421	0.3074
4	rs12640503	*LINC02283*	A	0.1096	0.0602	-	-	-	0.1096	0.0588	100.00%	0.0344	0.3319
11	rs117807585	*SORL1*	A	-0.2335	0.0322	-0.1895	0.0499	39.46%	-0.1137	0.0403	60.54%	-0.2559	0.2087
1	rs142802245	*SERINC2*	A	0.0944	0.0506	-	-	-	0.0944	0.0494	100.00%	-0.0478	0.2504
11	rs76367405	*SORL1*	A	0.2116	0.0501	0.2116	0.0487	100.00%	-	-	-	-0.1997	0.4440
10	rs138604348	*IPMK*	A	0.1805	0.0423	0.1805	0.0411	100.00%	-	-	-	-0.6043	0.9234
2	rs6733839	*SORL1*	T	0.1693	0.0159	0.1693	0.0154	100.00%	-	-	-	0.0109	0.1749
11	rs2101756	*SORL1*	A	0.0725	0.0407	-	-	-	0.0725	0.0398	100.00%	0.2922	0.1941
1	rs679515	*CR1*	T	0.1523	0.0184	0.1508	0.0183	95.31%	0.0795	0.0825	4.69%	0.4181	0.5033
1	rs6697005	*CR1*	A	-0.1416	0.0188	-0.1416	0.0183	100.00%	-	-	-	-0.0257	0.1844
6	rs1497525	*OR2B2*	A	0.1348	0.0294	0.1153	0.0353	65.46%	0.0745	0.0486	34.54%	-0.0328	0.3204
20	rs6014724	*CASS4*	A	0.1319	0.0267	0.1319	0.0259	100.00%	-	-	-	0.1091	0.1833
8	rs1532276	*CLU*	T	-0.1271	0.0146	-0.1266	0.0154	85.36%	-0.0564	0.0372	14.64%	-0.1920	0.2076
6	rs9275098	*HLA-DQB1*	T	-0.1237	0.0245	-0.1292	0.0262	82.93%	-0.0422	0.0578	17.07%	-0.1615	0.2847
11	rs3851179	*PICALM*	T	-0.1234	0.0140	-0.1198	0.0148	84.24%	-0.0618	0.0342	15.76%	-0.0997	0.1803
3	rs7618668	*CLEC3B*	A	-0.1220	0.0250	-0.1297	0.0258	88.94%	-0.0263	0.0732	11.06%	-0.1047	0.3193
2	rs35832505	*BIN1*	T	-0.1213	0.0196	-0.1213	0.0190	100.00%	-	-	-	-0.0733	0.3267
5	rs1001530	*FAM193B-DT*	A	-0.1210	0.0271	-0.1373	0.0342	59.65%	-0.0422	0.0416	40.35%	-0.1458	0.2133
11	rs1582763	*MS4A4E*	A	-0.1122	0.0145	-0.1232	0.0149	89.43%	-0.0088	0.0433	10.57%	-0.0496	0.2256

© 2022 Jung S-H et al. *JAMA Network Open.*

19	rs12151021	*ABCA7*	A	0.1071	0.0174	0.1071	0.0169	100.00%	-	-	-	0.0616	0.1696
14	rs8016766	*TEX22*	T	-0.1042	0.0253	-0.1042	0.0246	100.00%	-	-	-	-0.0825	0.1741
7	rs75045569	*EPHA1-AS1*	T	0.1040	0.0201	0.1040	0.0195	100.00%	-	-	-	0.2760	0.3120
14	rs74825460	*FERMT2, LOC105370500*	T	0.0984	0.0213	0.1200	0.0246	71.05%	0.0199	0.0385	28.95%	0.0686	0.2041
11	rs7926954	*LINC02705*	A	-0.0979	0.0143	-0.1126	0.0146	90.48%	0.0175	0.0450	9.52%	-0.2449	0.2210
14	rs12590273	*SLC24A4*	T	0.0974	0.0216	0.0935	0.0215	94.92%	0.0732	0.0929	5.08%	0.2885	0.4238
11	rs11605348	*NDUFS3, FAM180B*	A	-0.0968	0.0160	-0.0968	0.0155	100.00%	-	-	-	-0.1146	0.1862
19	rs3795065	*ABCA7*	T	-0.0968	0.0176	-0.0968	0.0171	100.00%	-	-	-	0.0350	0.2091
16	rs3752786	*MTSS2*	A	-0.0964	0.0215	-0.0964	0.0209	100.00%	-	-	-	-0.0816	0.2389
4	rs13101577	*LINC02498*	A	-0.0942	0.0210	-0.0922	0.0227	81.05%	-0.0445	0.0470	18.95%	-0.0779	0.2253
12	rs7962629	*C1S*	A	0.0922	0.0201	0.0867	0.0206	90.30%	0.0620	0.0629	9.70%	-0.1740	0.3250
6	rs9389138	*SLC2A12*	T	-0.0922	0.0221	-0.0978	0.0221	94.60%	0.0020	0.0925	5.40%	-0.0266	0.4607
2	rs6605277	*INPP5D*	A	0.0921	0.0209	0.0921	0.0203	100.00%	-	-	-	-0.0917	0.2333
6	rs3132963	*TSBP1, TSBP1-AS1*	A	-0.0919	0.0204	-0.0919	0.0198	100.00%	-	-	-	0.3855	0.5383
6	rs9270824	*HLA-DRB1*	T	0.0916	0.0175	0.0916	0.0170	100.00%	-	-	-	-0.0458	0.2038
8	rs28834970	*PTK2B*	T	-0.0909	0.0148	-0.0921	0.0153	87.95%	-0.0358	0.0413	12.05%	0.0735	0.2092
14	rs12590654	*SLC24A4*	A	-0.0906	0.0162	-0.0906	0.0157	100.00%	-	-	-	-0.1539	0.1779
17	rs61182333	*SCIMP, ZNF594-DT*	T	0.0874	0.0212	0.0874	0.0206	100.00%	-	-	-	0.0917	0.2568
2	rs17014923	*BIN1*	T	-0.0870	0.0184	-0.0863	0.0189	89.23%	-0.0404	0.0544	10.77%	0.1501	0.2680

© 2022 Jung S-H et al. *JAMA Network Open.*

16	rs12102869	*GPRC5B*	T	0.0870	0.0195	0.0870	0.0189	100.00%	-	-	-	0.1101	0.2073
11	rs11039165	*MADD*	A	-0.0865	0.0162	-0.0894	0.0158	99.16%	0.1114	0.1712	0.84%	0.0320	0.5361
6	rs4335021	*BTNL2*	T	0.0859	0.0147	0.0850	0.0155	84.97%	0.0394	0.0369	15.03%	0.0760	0.2141
10	rs12358692	*LOC105376412, LOC105376413*	T	0.0841	0.0159	0.0841	0.0154	100.00%	-	-	-	0.0305	0.1907
3	rs4574296	*LOC102723364*	A	0.0840	0.0200	0.1013	0.0223	76.33%	0.0124	0.0400	23.67%	0.0742	0.2047
6	rs9381563	*AL355353.1*	T	-0.0821	0.0152	-0.0821	0.0148	100.00%	-	-	-	-0.0686	0.2250
19	rs3865444	*CD33*	A	-0.0804	0.0163	-0.0804	0.0158	100.00%	-	-	-	0.0080	0.2181
1	rs61833519	*LOC343508*	T	0.0800	0.0191	0.0800	0.0186	100.00%	-	-	-	0.0175	0.2865
19	rs113704219	*TMEM259*	T	-0.0797	0.0193	-0.0764	0.0198	89.58%	-0.0467	0.0581	10.42%	-0.0807	0.3158
11	rs11230227	*MS4A4E*	A	0.0792	0.0157	0.0792	0.0153	100.00%	-	-	-	0.0373	0.1941
7	rs1989834	*LOC101928012*	T	-0.0790	0.0187	-0.0793	0.0188	93.74%	-0.0326	0.0727	6.26%	-0.0742	0.3104
14	rs1680666	*LOC107987210*	T	0.0789	0.0179	0.0789	0.0174	100.00%	-	-	-	-0.1542	0.1714
14	rs941648	*SLC24A4*	A	-0.0775	0.0152	-0.0829	0.0165	80.41%	-0.0241	0.0334	19.59%	-0.1510	0.1768
10	rs10748526	*TSPAN14*	T	-0.0773	0.0172	-0.0775	0.0179	87.23%	-0.0330	0.0468	12.77%	-0.0159	0.2447
13	rs9520713	*NALF1*	A	-0.0769	0.0166	-0.0730	0.0166	94.43%	-0.0616	0.0683	5.57%	0.3228	0.4013
17	rs2526378	*BZRAP1*	A	0.0767	0.0137	0.0717	0.0145	84.05%	0.0446	0.0333	15.95%	-0.0060	0.1759
11	rs598561	*SLC25A1P1*	A	0.0766	0.0140	0.0747	0.0143	90.35%	0.0410	0.0438	9.65%	0.1169	0.2505
4	rs11520553	*RNA5SP527*	T	0.0759	0.0179	0.0716	0.0181	92.43%	0.0558	0.0632	7.57%	0.4329	0.2900
15	rs72749540	*EFL1*	A	0.0758	0.0163	0.0700	0.0175	81.94%	0.0442	0.0373	18.06%	-0.0658	0.1986
8	rs7831810	*GULOP*	A	-0.0736	0.0138	-0.0765	0.0146	84.89%	-0.0248	0.0346	15.11%	-0.0790	0.1826
21	rs3017432	*ADAMTS1*	T	-0.0735	0.0155	-0.0735	0.0151	100.00%	-	-	-	-0.1106	0.1900

© 2022 Jung S-H et al. *JAMA Network Open.*

16	rs4985557	*MTSS2*	T	0.0734	0.0140	0.0652	0.0146	86.47%	0.0544	0.0369	13.53%	0.0193	0.1892
1	rs12118278	*KIF21B*	A	0.0730	0.0169	0.0798	0.0186	78.28%	0.0212	0.0353	21.72%	0.1106	0.1836
16	rs4782284	*IQCK*	A	0.0727	0.0175	0.0827	0.0189	81.46%	0.0126	0.0396	18.54%	0.0406	0.2162
7	rs60738304	*ZCWPW1*	A	-0.0711	0.0149	-0.0700	0.0160	81.74%	-0.0330	0.0339	18.26%	-0.1035	0.1740
19	rs8111708	*ELL*	A	-0.0704	0.0144	-0.0696	0.0151	86.16%	-0.0326	0.0377	13.84%	-0.1536	0.2000
5	rs11168036	*PFDN1*	T	0.0701	0.0135	0.0754	0.0143	84.35%	0.0182	0.0332	15.65%	0.1689	0.1767
6	rs12197146	*CD2AP*	T	0.0674	0.0148	0.0674	0.0144	100.00%	-	-	-	0.0377	0.2149
7	rs11769980	*EPHA1-AS1*	A	-0.0668	0.0145	-0.0684	0.0148	91.11%	-0.0222	0.0474	8.89%	-0.1691	0.2370
1	rs12030051	*EIF4G3*	A	0.0667	0.0146	0.0661	0.0153	86.41%	0.0307	0.0386	13.59%	-0.0354	0.2116
11	rs11607586	*UBASH3B*	T	0.0663	0.0153	0.0527	0.0164	82.76%	0.0569	0.0359	17.24%	0.2038	0.2008
11	rs12284553	*NTM, LOC107984413*	A	0.0661	0.0137	0.0617	0.0144	85.72%	0.0401	0.0353	14.28%	-0.2327	0.1993
10	rs7358283	*SH2D4B*	A	0.0652	0.0154	0.0656	0.0164	83.32%	0.0273	0.0367	16.68%	-0.0775	0.1774
11	rs12798036	*AP2A2*	T	-0.0638	0.0143	-0.0614	0.0152	83.77%	-0.0330	0.0345	16.23%	0.0975	0.1776
3	rs59930643	*ADCY5*	A	-0.0633	0.0147	-0.0586	0.0155	85.19%	-0.0390	0.0372	14.81%	0.1151	0.1903
6	rs1265759	*TSBP1, TSBP1-AS1*	T	-0.0630	0.0141	-0.0671	0.0150	83.17%	-0.0188	0.0333	16.83%	0.0298	0.1829
20	rs6076600	*RPL21P2*	A	0.0619	0.0150	0.0563	0.0154	89.24%	0.0469	0.0444	10.76%	-0.0426	0.2129
1	rs7536204	*USP24*	A	-0.0607	0.0141	-0.0641	0.0148	86.37%	-0.0170	0.0373	13.63%	0.0069	0.1864
2	rs1446445	*LOC105369165*	A	0.0572	0.0136	0.0557	0.0143	85.12%	0.0286	0.0342	14.88%	0.0751	0.1769
2	rs6722041	*FSIP2*	T	-0.0569	0.0135	-0.0636	0.0143	84.49%	-0.0090	0.0334	15.51%	-0.0768	0.1761
9	rs2480497	*LOC105376137*	T	-0.0568	0.0134	-0.0549	0.0142	84.53%	-0.0291	0.0332	15.47%	-0.0959	0.1711
3	rs614004	*CMTM7*	A	-0.0562	0.0134	-0.0538	0.0141	84.78%	-0.0302	0.0333	15.22%	-0.0478	0.1735

Statistical values were obtained from [1]a meta-GWAS, [2]previous studies [5, 6], and [3]our dataset 1.

© 2022 Jung S-H et al. *JAMA Network Open.*

[4]Proportion of SNP's weight of each cohort for the inverse-variance weighted model (METAL)

Abbreviations: NCGG, National Center for Geriatrics and Gerontology; IGAP, International Genomics of Alzheimer's Project; CHR, chromosome; SNP, single-nucleotide polymorphism; SE, standard error; EA, effect allele; PRS, polygenic risk score.

© 2022 Jung S-H et al. *JAMA Network Open.*

eTable 4. Characteristics of SNVs selected for Japanese-based PRS.

CHR	SNP	Nearest gene	EA	Japanese [6] (NCGG)	Korean	Japanese [6] (NCGG)		Korean (our dataset 1)	
				EAF[1]	EAF[1]	Beta[2]	SE[2]	Beta[3]	SE[3]
11	rs117807585	*SORL1*	A	0.2302	0.2167	-0.2619	0.0789	-0.2559	0.1480

Statistical values and [1]EAF were obtained from [2]a previous study [6] and [3]our dataset 1. Abbreviations: NCGG, National Center for Geriatrics and Gerontology; CHR, chromosome; SNP, single-nucleotide polymorphism; SE, standard error; EAF, effect allele frequency; PRS, polygenic risk score.

© 2022 Jung S-H et al. *JAMA Network Open.*

eTable 5. Predictive accuracy of PRSs derived from European, Japanese, and trans-ancestry meta-GWAS.

**ADD diagnosis**	**Dataset 1 (CU (n=479) vs. ADD (n=544))**			**Dataset 2 (CU (n=220) vs. ADD (n=159))**		
Derivation GWAS	[1]OR (95% CI)	[1]*P*	[2]Nagelkerke's R^2	[1]OR (95% CI)	[1]*P*	[2]Nagelkerke's R^2
European GWAS (IGAP)	1.95 (1.40–2.72)	<.001	0.020	1.85 (1.05–3.32)	.04	0.026
Japanese GWAS (NCGG)	2.71 (1.16–6.40)	.02	0.006	3.26 (0.71–15.55)	.13	0.006
Trans-ancestry meta-GWAS	1.69 (1.31–2.19)	<.001	0.023	2.09 (1.09–4.04)	.03	0.032

[1]The OR and *P* values for PRS were calculated using multivariable logistic regression model after adjusting sex, age, education year, four PCs of genetic data, and *APOE* ε4 status.

[2]Variance explained by PRS.

Abbreviations: CU, cognitively unimpaired; ADD, Alzheimer's disease dementia; OR, odds ratio; CI, confidence interval; GWAS, genome-wide association study; PRS, polygenic risk score; IGAP, European International Genomics of Alzheimer's Project; NCGG, National Center for Geriatrics and Gerontology; PC, principal component.

© 2022 Jung S-H et al. *JAMA Network Open.*

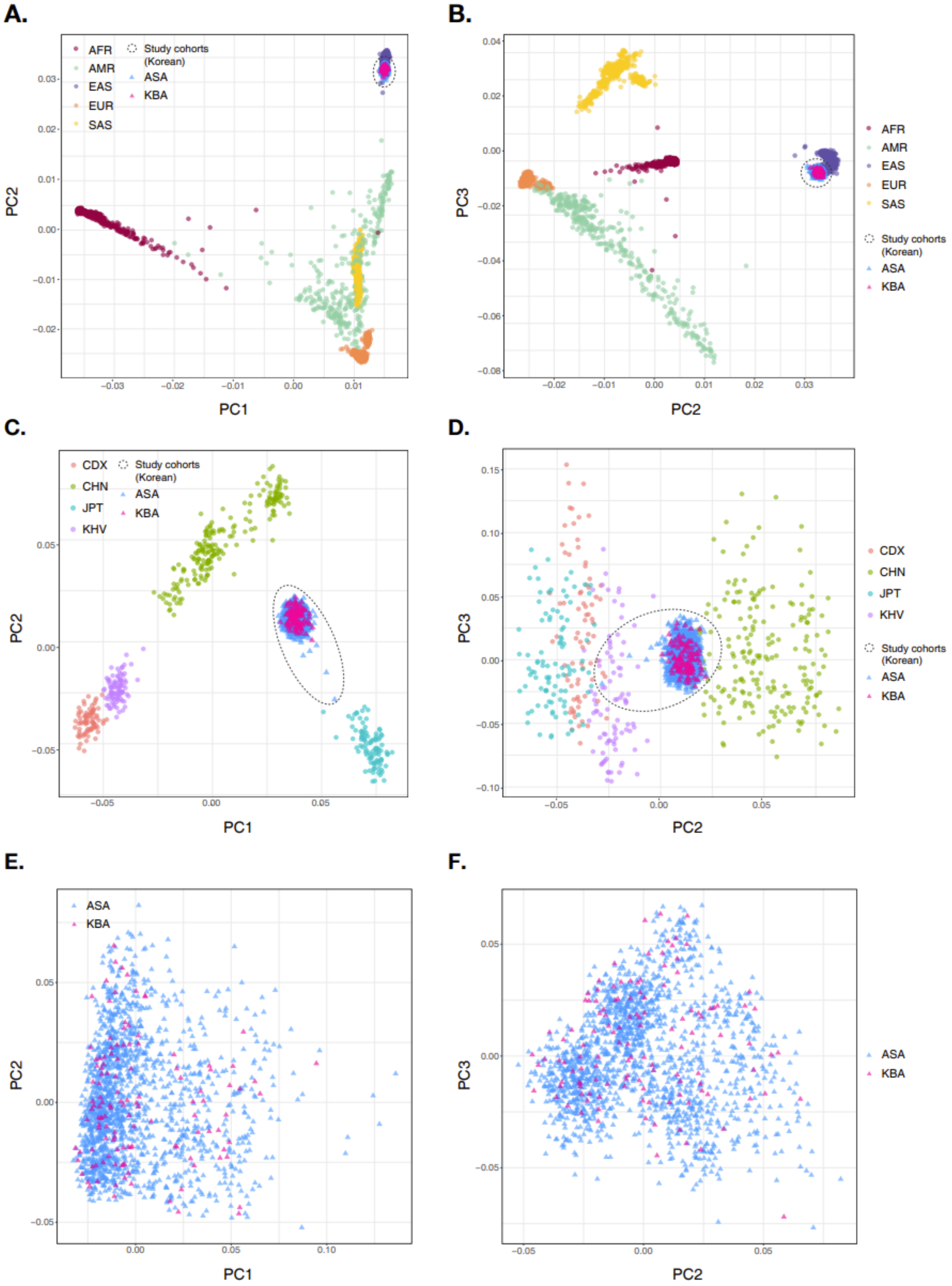

© 2022 Jung S-H et al. *JAMA Network Open.*

eFigure 1. Principal component analysis comparison of the Korean study cohorts and the 1000 Genomes Project populations. The first three PCs of genetic ancestry are presented. Each dot represents an individual from the ethnic group represented by different colours. **(A), (B)** PCs of the 1000 Genomes Project dataset (circle; AFR, AMR, EAS, EUR, and SAS) and the current study cohorts (triangle; ASA and KBA). **(C), (D)** PCs of the 1000 Genomes Project East Asian dataset (circle; CDX, CHN, JPT, and KHV) and the current study cohorts (triangle; ASA and KBA). **(E), (F)** PCs of ASA and KBA samples from independent PCA without projection to the 1000 Genomes Project. Abbreviations: PC, principal component; ASA, Illumina Asian Screening Array BeadChip (Korean study cohorts); KBA, Affymetrix Axiom Korea Biobank Array (Korean study cohorts); AFR, African; AMR, American; EAS, East Asian; EUR, European; SAS, South Asian; CDX, Chinese Dai in Xishuangbanna, Chinese; CHN, Han Chinese in Beijing, Chinese; JPT, Japanese in Tokyo, Japan; KHV, Kinh in Ho Chi Minh City, Vietnam.

© 2022 Jung S-H et al. *JAMA Network Open.*

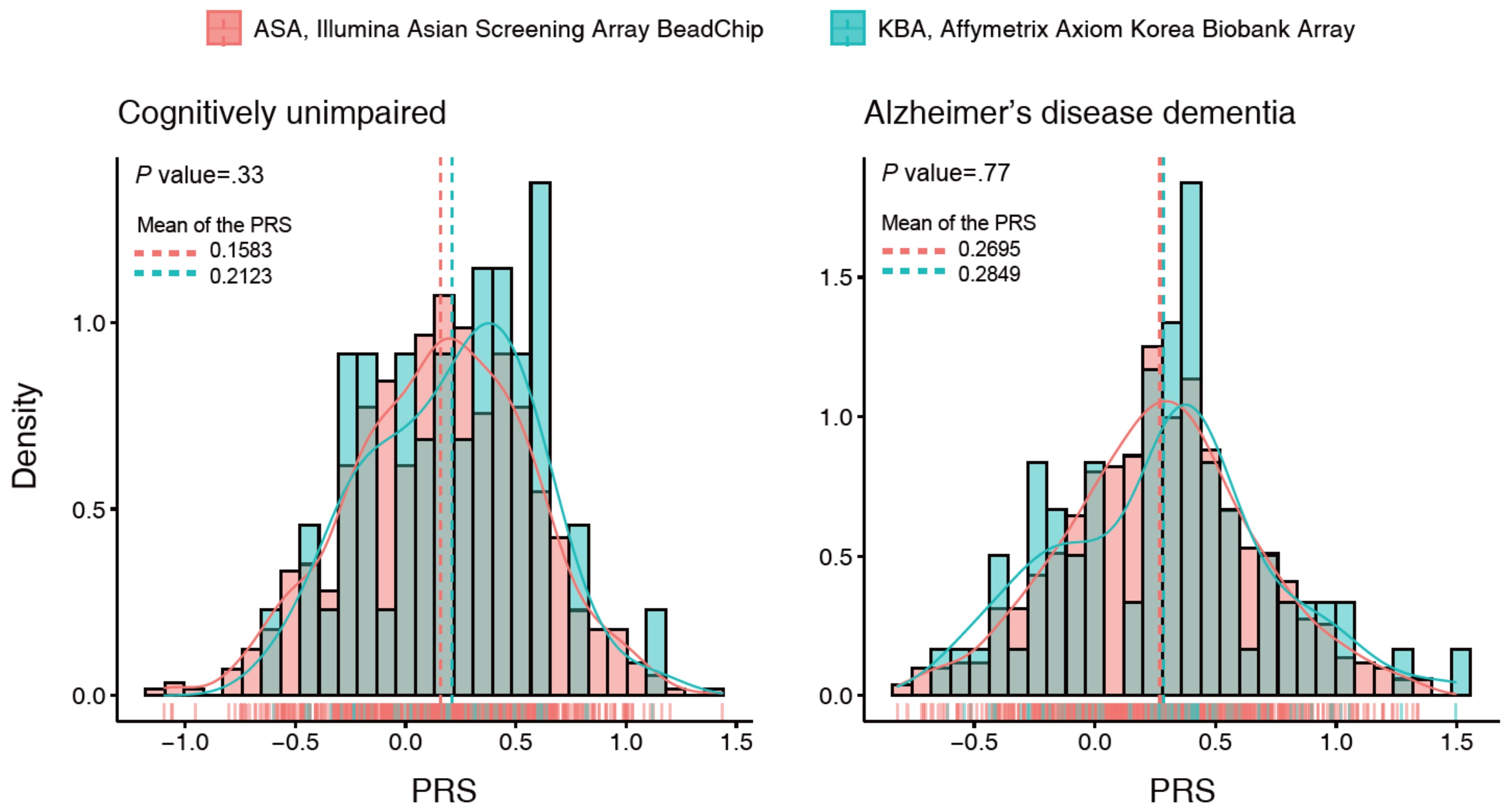

eFigure 2. PRS distributions among the study participants according to the genotyping arrays. Abbreviations: ASA, Illumina Asian Screening Array BeadChip; KBA, Affymetrix Axiom Korea Biobank Array; PRS, polygenic risk score.

© 2022 Jung S-H et al. *JAMA Network Open.*

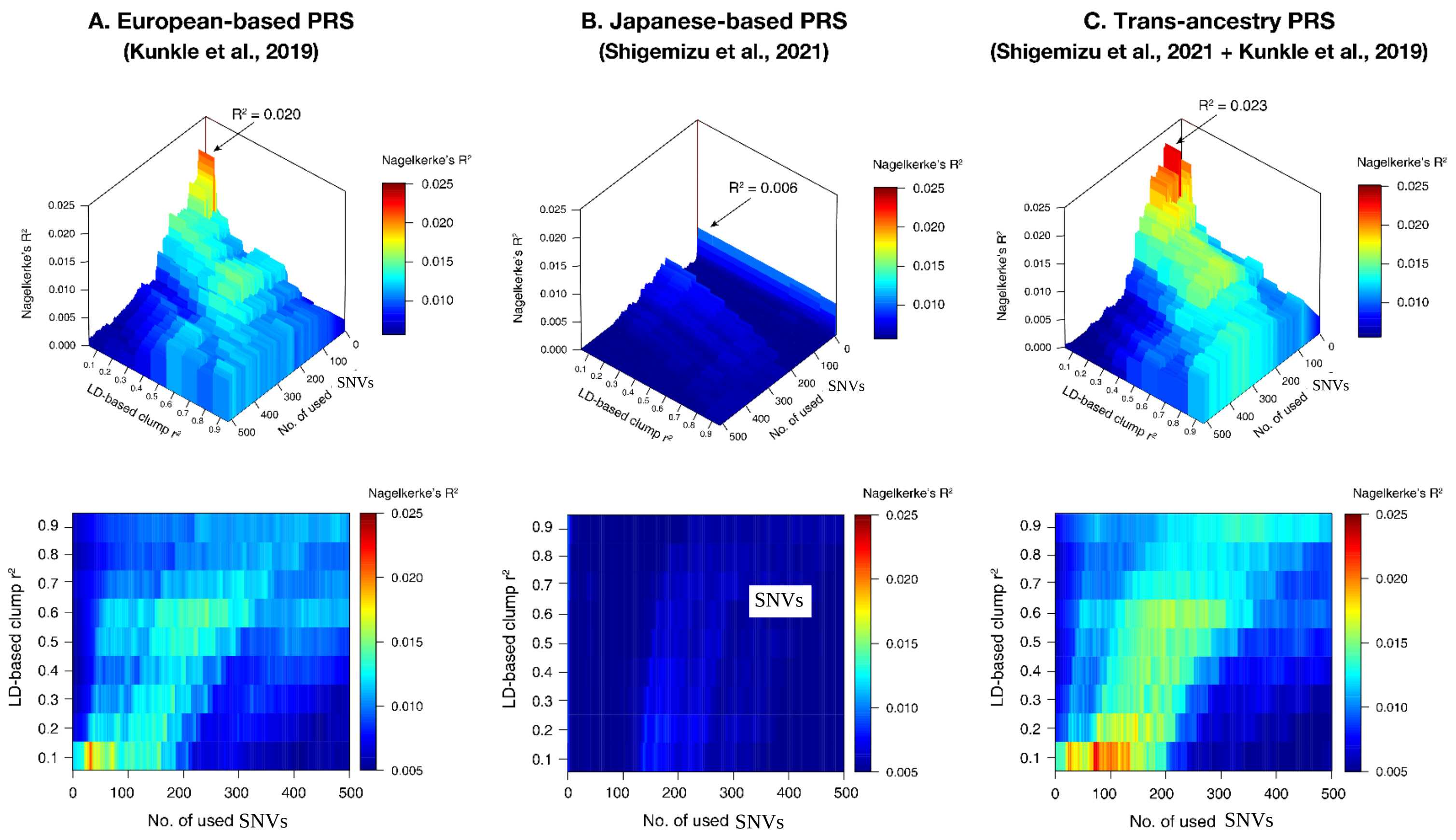

eFigure 3. Distribution plots for Nagelkerke's R^2 values of each population-based PRS across the SNP selection thresholds.

Abbreviations: PRS, polygenic risk score; SNP, single-nucleotide polymorphism; LD, linkage disequilibrium.

© 2022 Jung S-H et al. *JAMA Network Open.*

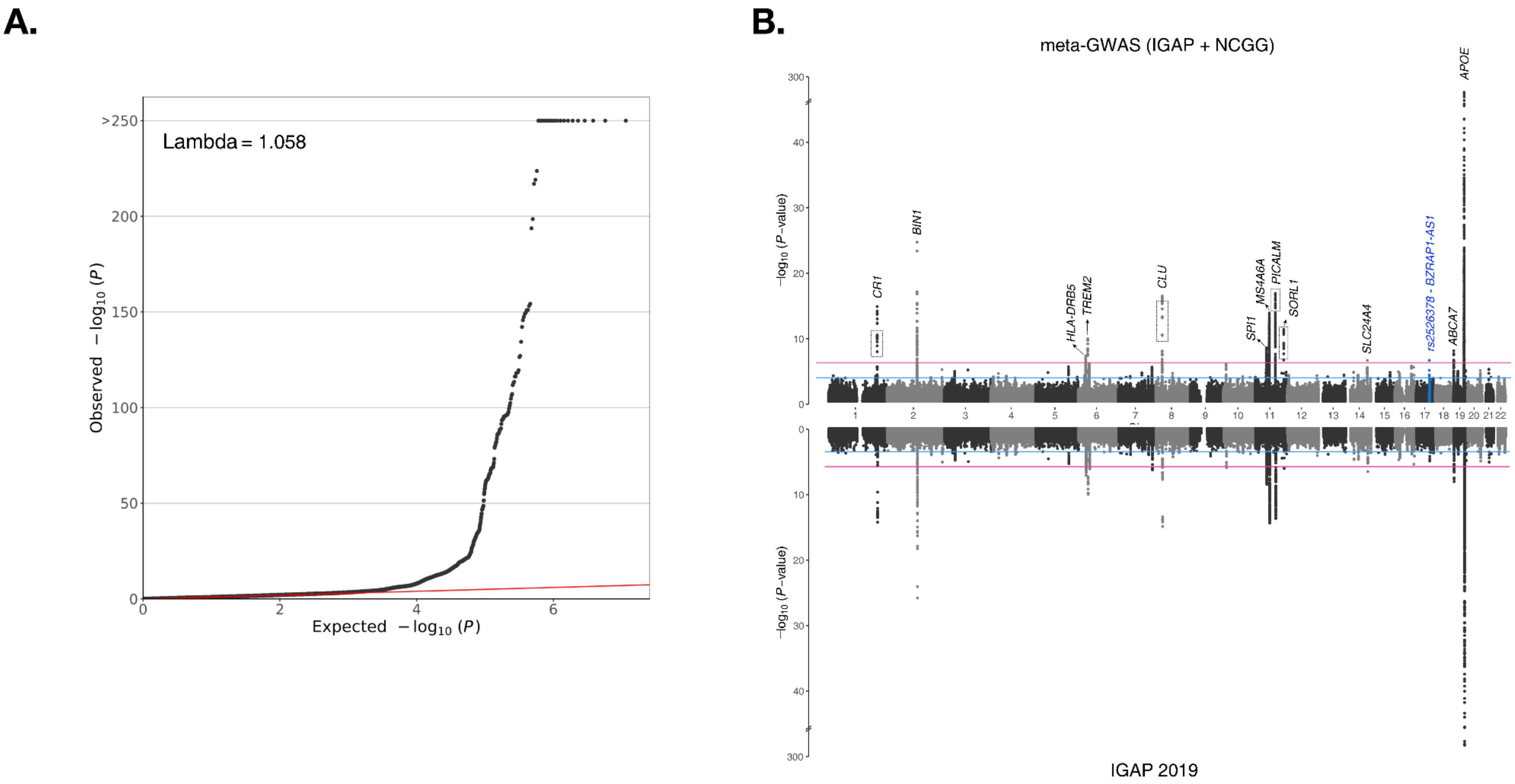

eFigure 4. (A) Quantile-quantile plot (B) Miami plot for the trans-ancestry meta-GWAS. The red and blue lines indicate the genome-wide significance level (P=5.0×10^{-8}) and the genome-wide suggestive level (P=1.0×10^{-5}), respectively. A locus near rs2526378 on chromosome 17 were newly identified in the trans-ancestry meta-GWAS.

Abbreviations: IGAP, International Genomics of Alzheimer's Project; NCGG, National Center for Geriatrics and Gerontology.

© 2022 Jung S-H et al. *JAMA Network Open.*

eReferences

[1] Price, A. L., Patterson, N. J., Plenge, R. M., Weinblatt, M. E., Shadick, N. A., & Reich, D. (2006). Principal components analysis corrects for stratification in genome-wide association studies. *Nature genetics*, **38**(8), 904-909.

[2] Manichaikul, A., Mychaleckyj, J. C., Rich, S. S., Daly, K., Sale, M., & Chen, W. M. (2010). Robust relationship inference in genome-wide association studies. *Bioinformatics*, **26**(22), 2867-2873.

[3] Villemagne, V. L., Ong, K., Mulligan, R. S., Holl, G., Pejoska, S., Jones, G., ... & Rowe, C. C. (2011). Amyloid imaging with 18F-florbetaben in Alzheimer disease and other dementias. *Journal of Nuclear Medicine*, **52**(8), 1210-1217.

[4] Curtis, C., Gamez, J. E., Singh, U., Sadowsky, C. H., Villena, T., Sabbagh, M. N., ... & Salloway, S. (2015). Phase 3 trial of flutemetamol labeled with radioactive fluorine 18 imaging and neuritic plaque density. *JAMA neurology*, **72**(3), 287-294.

[5] Kunkle, B. W., Grenier-Boley, B., Sims, R., Bis, J. C., Damotte, V., Naj, A. C., ... & Rotter, J. I. (2019). Genetic meta-analysis of diagnosed Alzheimer's disease identifies new risk loci and implicates Aβ, tau, immunity and lipid processing. *Nature genetics*, **51**(3), 414-430.

[6] Shigemizu, D., Mitsumori, R., Akiyama, S., Miyashita, A., Morizono, T., Higaki, S., ... & Ozaki, K. (2021). Ethnic and trans-ethnic genome-wide association studies identify new loci influencing Japanese Alzheimer's disease risk. *Translational psychiatry*, **11**(1), 1-10.

© 2022 Jung S-H et al. *JAMA Network Open.*
